# Evaluating surface water for genomic surveillance of community-linked antimicrobial resistance in ESBL-producing *Klebsiella pneumoniae*

**DOI:** 10.1016/j.envadv.2026.100744

**Published:** 2026-10

**Authors:** Etando Ayukafangha, Joshua Mbanga, Arshad Ismail, Sabiha Y. Essack, Akebe Luther King Abia

**Affiliations:** a Antimicrobial Research Unit, College of Health Sciences, University of KwaZulu-Natal, Durban, South Africa; b Department of Applied Biology and Biochemistry, National University of Science and Technology, P. Bag AC939 Ascot, Bulawayo, Zimbabwe; c Sequencing Core Facility, National Institute for Communicable Diseases, National Health Laboratory Service, Johannesburg, 2131, South Africa

**Keywords:** Surveillance, Antimicrobial resistance, Surface water, Epidemiology, Public health

## Abstract

A major limitation in wastewater-based epidemiology is its application in communities without centralised sewage systems necessitating alternative approaches.

We evaluated surface water as an alternate antimicrobial resistance (AMR) monitoring interface by comparing the resistome, virulome, and phylogeny of extended-spectrum β-lactamase-producing *Klebsiella pneumoniae* (ESBL-Kp) from surface water (SW), healthy residents (HR), and clinical (CL) settings.

Ninety-six (SW = 32, HR = 32, CL = 32) ESBL-Kp isolates collected over eight-months underwent comparative genomic analysis of antibiotic resistance genes (ARGs), virulence determinants, multilocus sequence types, and phylogenetic relatedness.

Sixteen sequence types (STs) were identified across all sources. ST983 was shared by all sources, ST25 and ST219 were exclusive to SW-HR, and ST152 to SW-CL. More SW (93.8%) isolates shared STs with HR than with CL (9.4%). Phylogenomic analysis was lineage-driven rather than source-structured, with SW isolates forming stronger subclades with HR through ST25 and ST219. Resistome composition showed substantial overlap with differences (*p* = 0.003). The dominant β-lactamase ARG in all sources was *bla*_CTX-M-15_, alongside non-β-lactam *sul2* and *dfrA14*. Pairwise exclusive ARGs totalled 20 (CL-HR), seven (SW-HR), and two (SW-CL); source-restricted ARGs totalled fourteen, nine, and none for CL, HR, and SW, respectively. SW-HR. Virulome overlap was driven by ST25/KL2 and ST219/KL114, a limited SW-CL link was observed through ST152/KL149, and source-restricted lineages were concentrated mainly in clinical and healthy resident isolates.

SW shared major ESBL-Kp AMR features with both HR and CL, but distribution differed across sources. SW more closely resembled HR, supporting its use as a complementary tool rather than a standalone approach for AMR surveillance.

## Highlights

• Surface water captured dormant ESBL-Kp lineages in community and clinical sources.
• Surface water AMR profile most closely resembled healthy resident isolates.
• *bla*_CTX-M-15_, *sul2* and *dfrA14* predominated across isolate from all sources.
• ESBL-*K pneumoniae* from healthy residents showed the highest genomic diversity.
• Surface water complements AMR surveillance when wastewater systems are lacking.

## Graphical abstract

**Figure:**
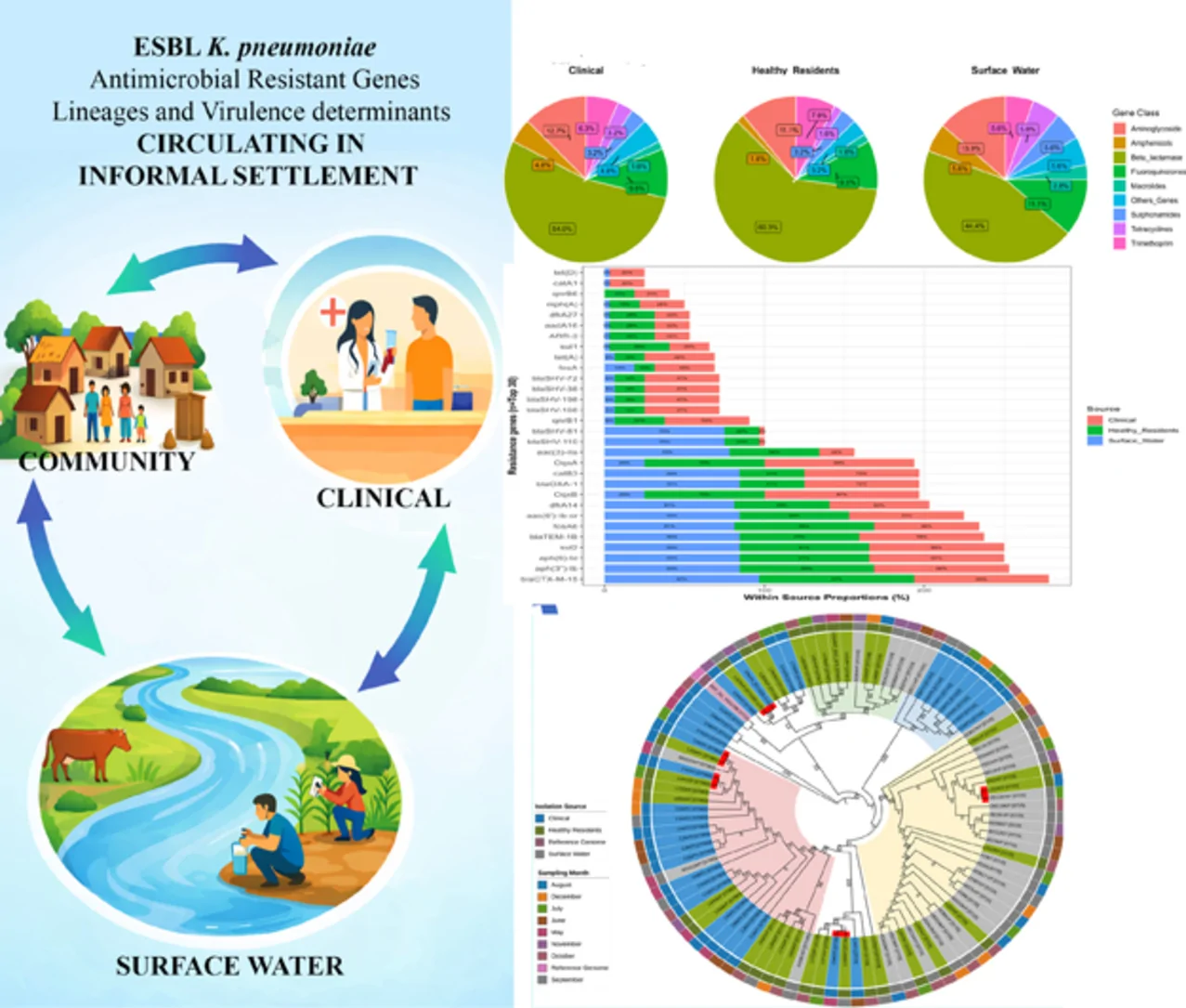

## 1 Introduction

Antimicrobial resistance (AMR) is a major public-health threat that compromises the effective treatment of infections worldwide. Its impact is particularly severe in low- and middle-income countries (LMICs), where a high infectious disease burden often intersects with limited diagnostic capacity, weak laboratory infrastructure, fragmented health information systems and uneven access to routine microbiological testing (Iskandar et al., 2021; Ondoa et al., 2025). Conventional AMR surveillance is largely based on clinical isolates obtained through healthcare systems and therefore predominantly represents individuals who access formal care, have appropriate specimens collected and are linked to functioning laboratory-reporting systems (Holt et al., 2025). Consequently, facility-based surveillance may underrepresent resistance circulating among asymptomatic community populations and does not adequately capture environmental reservoirs that may contribute to AMR persistence and dissemination.

These limitations have increased interest in environmental surveillance as a complementary component of AMR monitoring. Wastewater-based epidemiology has gained particular attention because wastewater can integrate microbial and resistance signals from large sewered catchment populations. Large-scale wastewater metagenomic studies have demonstrated the value of sewage for mapping antimicrobial-resistance gene distributions across countries, while subsequent studies have identified associations between sewage resistomes and clinical resistance patterns (Hendriksen et al., 2019; Karkman et al., 2020). However, wastewater surveillance is most readily implemented where waste from a defined population is collected through centralised sewerage and can be sampled at common points, such as wastewater-treatment-plant influent. Its applicability may therefore be limited in LMIC and resource-constrained settings where sewer connectivity is incomplete or absent and human waste enters pit latrines, septic systems, open drains, stormwater channels, canals, runoff and surrounding surface waters (Shrestha et al., 2021; Truyens et al., 2025; WHO/UNICEF, 2023).

Surface water may represent a contextually relevant environmental surveillance matrix in communities without comprehensive sewage infrastructure. In such settings, rivers and streams may receive diffuse inputs from households, informal sanitation systems, stormwater runoff, healthcare-associated activities and direct waste discharge, potentially integrating AMR signals from multiple human-associated sources (Meradji et al., 2025). Previous studies have demonstrated the occurrence of multidrug-resistant and ESBL-producing Enterobactera,les in surface waters affected by wastewater discharge, poor sanitation and other anthropogenic activities, including clinically relevant resistance genes and bacterial lineages (Bartley et al., 2019; Blaak et al., 2015; Guyomard-Rabenirina et al., 2017). However, these studies primarily established surface water as a receiving environment or reservoir for AMR by comparing surface water and wastewater compartments or assessing anthropogenic contamination. Evidence remains limited on whether surface-water isolates reflect the genomic resistance characteristics, circulating lineages and phylogenetic relationships observed among spatially connected community-carriage and clinical populations.

The need to evaluate such environmental matrices is particularly relevant in sub-Saharan Africa, where AMR surveillance remains uneven and many populations are not comprehensively represented by routine facility-based systems. South Africa has made important progress through national AMR surveillance initiatives aligned with global frameworks such as GLASS, particularly within public-sector hospital and laboratory networks (Engler et al., 2021; NDoH, 2024). Nevertheless, surveillance coverage may remain constrained in some rural and informal settlement settings by unequal access to healthcare, microbiological testing and centralised sanitation infrastructure (Mataboge et al., 2016; NDoH, 2024; Stats SA, 2025). In 2024, Stats SA reported that 76.3% of South Africans had safely managed sanitation, indicating that nearly one in four still lacked such access, with shared sanitation facilities particularly common in urban areas. In such communities where domestic and human waste may enter nearby rivers and streams, surface water may constitute a more relevant environmental receiving matrix than wastewater collected through formal sewer networks. Its capacity to reflect AMR circulating in connected human populations, however, requires direct evaluation.

Within this context, ESBL-producing *K. pneumoniae* is a clinically relevant target organism for evaluating whether surface water reflects human-associated AMR. *K. pneumoniae* is an opportunistic gut-associated pathogen and an important cause of healthcare- and increasingly community-associated infections. It also has a flexible accessory genome and frequently carries acquired resistance determinants on plasmids and other mobile genetic elements, making ESBL-producing strains useful for tracking multidrug-resistant Enterobacterales across human and environmental compartments (Caneiras et al., 2019). Its public health importance is reinforced by the 2024 WHO Bacterial Priority Pathogens List, which ranks carbapenem-resistant *K. pneumoniae* among the highest-priority bacterial threats for research, development, and public health action (WHO, 2024). Beyond clinical settings, resistant *K. pneumoniae* and members of the *K. pneumoniae* species complex have been recovered from wastewater and surface waters, with genomic studies showing that wastewater pathways can contribute to the persistence and environmental release of clinically relevant resistant lineages (Davidova-Gerzova et al., 2024; King et al., 2020; Verburg et al., 2024).

This study evaluated whether surface water can serve as an alternative matrix for human AMR surveillance in low-resource settings by comparing the resistome, virulome, and phylogenetic relatedness of ESBL-producing *K. pneumoniae* isolates from healthy residents of an informal settlement, surface water flowing through the community, and a proximate healthcare facility.

## 2 Materials and methods

### 2.1 Ethical statement

The protocol was approved by the Biomedical Research Ethics Committee, University of KwaZulu-Natal (BREC/00003640/2021), the KwaZulu-Natal Department of Health Provincial Health Research Ethics Committee (NHRD Ref. KZ_202203_023), and the National Health Laboratory Service (NHLS Ref. PR2225862). Written informed consent was secured from all community volunteers prior to study enrolment.

### 2.2 Study site and sample collection

This study was conducted in the uMgungundlovu District Municipality (UMDM), KwaZulu-Natal, South Africa. It was part of a broader longitudinal investigation evaluating the phenotypic and genomic characteristics of ESBL-producing *K. pneumoniae* across three interconnected sources: surface water, healthy community residents, and clinical settings. This sub-study examined concordance in genomic antimicrobial resistance profiles across these sources at higher resolution, with a particular focus on comparative analysis of the resistome, virulome and phylogenetic relationships among selected isolates.

Community samples consisted of stool specimens collected from healthy residents in an informal settlement. Clinical isolates were obtained from bacteriological cultures of various specimen types such as stool, urine, blood, cerebrospinal fluid, pus, and other body fluids from hospitalised adult patients (>18 years old), processed by the Medical Microbiology service of the NHLS serving the UMDM. Surface water samples were collected from the Kwapata River, which drains into the Msunduzi River, a major tributary of the uMngeni River flowing across the district as previously described (Ayukafangha et al., 2026).

### 2.3 *K. pneumoniae* identification, ESBL confirmation and antimicrobial susceptibility testing (AST)

Eighty surface-water samples, 1978 stool specimens, and 2154 clinical cultures yielded 462, 1667, and 244 presumptive *K. pneumoniae* isolates, respectively, following phenotypic isolation and identification on Simmons Citrate Agar supplemented with 1% (w/v) myo-inositol (SCAI) (Oxoid™, Basingstoke, United Kingdom; Sigma-Aldrich, St. Louis, Missouri, USA). Presumptive isolates were subjected to DNA extraction using the boiling method as previously described (Mbanga et al., 2020). Molecular confirmation was performed using qualitative real-time PCR targeting the *phoE* housekeeping gene. Briefly, each 10-µL reaction contained 5 µL Luna® Universal qPCR Master Mix (New England Biolabs, Ipswich, MA, USA), 1 µL nuclease-free water, 0.5 µL each of the forward and reverse primers, and 3 µL of template DNA. The *phoE*-specific primers were phoE-F (5′-TGCCCAGACCGATAACTTTA-3′) and phoE-R (5′-CTGTTTCTTCGCTTCACGG-3′) (Sun et al., 2010). Amplification comprised an initial denaturation at 94°C for 2 min, followed by 35 cycles of denaturation at 94°C for 30 s, annealing at 55°C for 30 s, and extension at 72°C for 30 s, followed by melting-curve analysis. Isolates showing amplification and a single melt peak corresponding to that of the *K. pneumoniae* ATCC 35657 positive control were considered *phoE*-positive. A no-template control was included in each run to detect possible contamination.

The PCR-confirmed *K. pneumoniae* were then subjected to ESBL screening and AST studies, yielding 60, 74, and 37 ESBL producers in surface water, healthy residents, and clinical sources, respectively, as per the ESBL screening and confirmation protocol previously described (Ayukafangha et al., 2026). Briefly, isolates were screened for ESBL production using CHROMID® ESBL agar (bioMérieux, Marcy l’Étoile, France). Phenotypic confirmation was performed using the double-disk synergy test, with cefotaxime (30 µg) and ceftazidime (30 µg) disks positioned 20 mm from a centrally placed amoxicillin/clavulanic acid disk (20/10 µg). An increase of ≥5 mm in the inhibition-zone diameter around either cephalosporin disk was interpreted as a positive ESBL result. Antimicrobial susceptibility testing was performed using the VITEK® 2 system with the AST-N256 card (bioMérieux, Marcy l’Étoile, France) for Gram-negative bacteria, following the manufacturer’s instructions. Results were interpreted according to the Clinical and Laboratory Standards Institute guidelines (CLSI, 2021).

Confirmed ESBL-producing *K. pneumoniae* isolates from each source underwent genomic DNA extraction using the GenElute™ Bacterial Genomic DNA Kit (Sigma-Aldrich, St Louis, Missouri, USA). DNA concentration and purity were assessed using a NanoDrop microvolume spectrophotometer (Thermo Fisher Scientific, Waltham, Massachusetts, USA) at a 260/280 nm ratio in accordance with the manufacturer’s protocol.

### 2.4 Whole-genome sequencing (WGS) and selection of study isolates

The extracted DNA of confirmed ESBL-producing *K. pneumoniae* isolates underwent whole-genome sequencing (WGS) at the National Institute for Communicable Diseases (NICD) Sequencing Core Facility. Sequenced isolates were screened for non-*K. pneumoniae* contamination, after which 32 clinical isolates remained. As this represented the smallest source group, all 32 clinical isolates were retained for comparative analysis. To obtain balanced source groups, 32 isolates were randomly selected from each of the larger healthy-resident and surface-water isolate pools using month-stratified sampling, with four isolates selected per source per month. This balanced, month-stratified selection reduced disproportionate representation of the larger isolate pools while preserving temporal coverage. The final comparative genomic dataset, therefore, comprised 96 ESBL-producing *K. pneumoniae* isolates: 32 clinical, 32 healthy-resident, and 32 surface-water isolates.

### 2.5 Bioinformatic analyses

Multiplexed paired-end libraries were prepared using the Illumina DNA Prep Kit (Illumina, San Diego, CA, USA), according to the manufacturer’s instructions. Library quality and fragment size distribution were assessed using an Agilent TapeStation (Agilent Technologies, Santa Clara, CA, USA), and library quantification was performed using a Qubit 4.0 Fluorometer (Thermo Fisher Scientific, Waltham, MA, USA). Standardised libraries were pooled at 4 nM to achieve approximately 100 × coverage per sample and sequenced at NICD (Johannesburg, South Africa) on the Element Biosciences AVITI and Illumina NextSeq 1000/2000 platforms to generate 2 × 150 bp paired-end reads. Raw sequence reads were quality-checked using FASTQC v0.12.0 (Andrews, 2010) and trimmed with Sickle v1.33 (https://github.com/najoshi/sickle) before de novo assembly with SKESA v2.4.0 (https://github.com/ncbi/SKESA). Draft assemblies were included in downstream analyses if they had a minimum read depth of 30 ×, at least 95% estimated genome coverage, and an assembled genome size within the expected range of 4.4-6.5 Mb. Contigs shorter than 200 bp were removed. The resulting quality-filtered draft genomes were submitted to NCBI GenBank under BioProject PRJNA1253337, and isolate-specific Genome and BioSample accession numbers, together with assembly-quality metrics, are provided in Supplementary Material S1.

To characterise the resistome, acquired antimicrobial resistance genes, point mutations, and disinfectant resistance genes were identified using ResFinder v4.7.2 (http://genepi.food.dtu.dk/resfinder) (Bortolaia et al., 2020) with default parameters, while resistance mechanisms and associated genes were further examined using the Comprehensive Antimicrobial Resistance Database (CARD) (https://card.mcmaster.ca) (Alcock et al., 2023). Sequence types were assigned using MLST v2.0.

(https://bitbucket.org/genomicepidemiology/mlst). To characterise the virulome and surface antigens, virulence determinants together with capsular (K) and lipopolysaccharide (O) loci were identified using Kleborate v3.2.4 with the integrated Kaptive v3 module (Lopes et al., 2025). In addition to broader virulome characterisation, isolates were specifically screened for the major hypervirulent *K. pneumoniae*-associated markers aerobactin (*iuc*), salmochelin (*iro*), and the hypermucoidy regulators *rmpA* and *rmpA2*. Phylogenomic analysis was performed on isolates across sources together with a reference genome (*K. pneumoniae* HS11286, ST11) from BV-BRC. All genomes were retrieved, annotated, and processed using the Bacterial and Viral Bioinformatics Resource Center (BV-BRCB; https://www.bv-brc.org/). Maximum-likelihood phylogenetic reconstruction was carried out within BV-BRC, and resulting trees were visualised in Interactive Tree of Life (iTOL; https://itol.embl.de). To quantify relatedness within sequence types shared across sources, assembled genomes underwent lineage-specific core-genome multilocus sequence typing (cgMLST) analysis using BV-BRC. Pairwise core-genome allelic distances were summarised by source pair, with ≤10 allelic differences used as an indicative threshold for close relatedness based on previous *K. pneumoniae* studies (Zhou et al., 2017). Annotation, customisation, and incorporation of associated metadata were performed in R (Zhou et al., 2023)

### 2.6 Statistical analyses

Statistical analyses were performed in R version 4.5.0 using RStudio version 2025.09.0.387. Genomic determinants were summarised at the isolate level as frequencies, proportions, andpresence-absence variables, as appropriate. Associations between isolate source and categorical genomic variables were assessed using Pearson’s chi-square test implemented with the “chisq.test” function in the R stats package. Where the assumptions of the chi-square test were not met because of sparse expected cell counts, Fisher’s exact test was applied using the “fisher.test” function, with Monte Carlo simulation for larger contingency tables where required. Standardised residuals were examined to identify categories contributing to statistically significant associations. *P*-values of multiple comparisons were adjusted using the Benjamini-Hochberg false discovery rate procedure (Benjaminit and Hochberg, 1995).

Diversity in the distributions of resistance genes, resistance mechanisms, and virulence-related determinants was summarised using the Shannon diversity index and Pielou’s evenness (Pielou, 1966; Shannon, 1948), implemented using established functions in the vegan package. Principal component analysis (PCoA) was performed on centred and scaled isolate-by-gene presence-absence matrices using the “prcomp” function in the R stats package to visualise similarities in resistome composition among sources (Jolliffe and Cadima, 2016).

Differences in multivariate resistome composition among sources were tested using permutational multivariate analysis of variance based on Bray-Curtis dissimilarities, implemented with the "adonis2" function in the vegan package (Anderson, 2001; Oksanen et al., 2025). Homogeneity of multivariate dispersion was evaluated using “betadisper” followed by permutation testing to support interpretation of the PERMANOVA results. Statistical significance was defined as *p* < 0.05. Data visualisations, including bar plots, ordinations, and heatmaps, were generated using ggplot2 and ComplexHeatmap in R (Gu et al., 2016; Wickham, 2016).

## 3 Results

### 3.1 Genomic features of ESBL-Kp isolates

Assembly metrics for the 96 quality-filtered draft genomes are summarised in Supplementary Material S1. Genome sizes ranged from 5.31 to 5.66 Mb, with GC contents ranging from 56.38% to 57.81%. Draft assemblies comprised 38–199 contigs, with N50 values ranging from 22.27 to 477.31 kb and L50 values from 3 to 19. Genome annotation identified 5,124-6,427 coding sequences, 73-90 tRNA genes, and 2-14 rRNA genes per genome. BV-BRC quality assessment yielded coarse-consistency scores of 97.5-99.6% and fine-consistency scores of 91.0-98.7%.

### 3.2 MLST and phylogenetic relationships

MLST analysis identified 16 distinct sequence types (STs) across all sources. Only one sequence type, ST983, was shared across surface water (SW), healthy residents (HR), and clinical (CL) isolates. Two STs (ST25 and ST219) were exclusive to SW and HR, and one (ST152) to SW and CL. Five ST were unique to CL, four to HR, and one to SW, as shown in Fig. 1a.

**Fig. 1:**
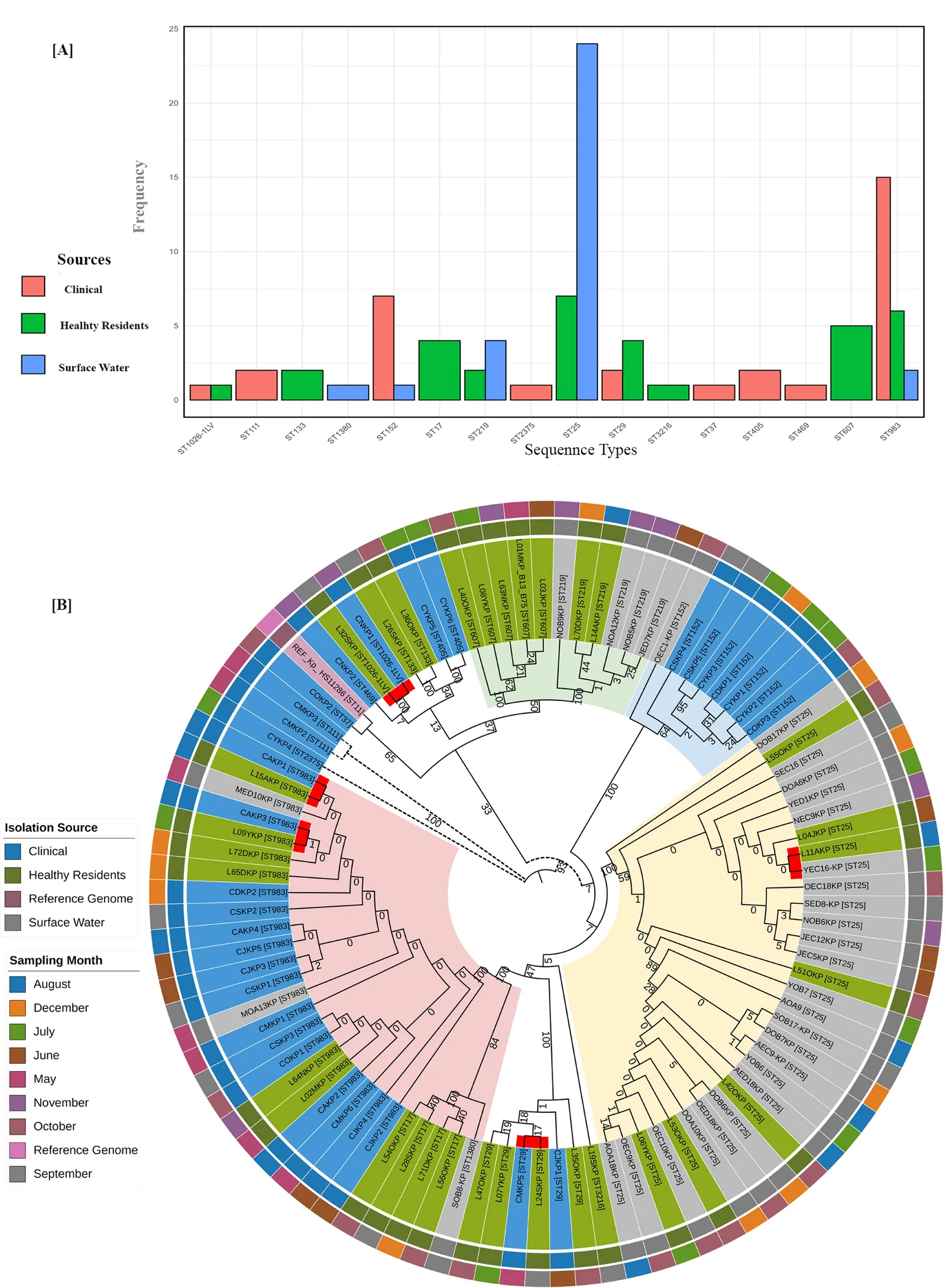
[A] Distribution of sequenced types across isolates sources. [B] Phylogenetic tree shows the relationships among study isolates based on a maximum-likelihood analysis. Isolate IDs and sequence types (STs) are displayed at the tips. Tip background colours indicate source of isolation: clinical (blue), healthy residents (dark green), and surface water (grey). Concentric metadata rings, shown from inner to outer, represent isolation source and sampling month. The four major mixed-source clades are shaded (yellow, pink, green, and sky-blue), within source clades are highlighted in sky-bue, and tight or sister cross-source clustering is highlighted in red. Internal node values indicate bootstrap support (0–100).

Consistent with this distribution, codon-based maximum-likelihood phylogenetic analysis of the 96 isolates and one reference genome (*K. pneumoniae* HS11286, ST11) revealed a population structure primarily shaped by clonal lineage rather than by source of isolation (Fig. 1b). The majority of isolates (*n* = 94) clustered within a single dominant clade (BS = 93%), while two independently divergent lineages, both exclusively clinical, branched outside this clade (the dotted black lines).

Within the dominant clade, several well-supported substructures were evident. The largest was an ST25 subclade (yellow clade) (BS = 100%, *n* = 31), composed predominantly of SW isolates (*n* = 24) together with a smaller number of HR isolates (*n* = 7) and notably lacking any clinical isolate. A second major lineage centred on ST983 (Pink clade) grouped isolates from all three sources (15 CL, 6 HR, and 2 SW) without clear internal separation by source, representing the most prominent source connectedness. A third lineage, defined by ST152 (sky-blue subclade, BS = 100%, *n* = 8), consisted mainly of clinical isolates (*n* = 7) connected SW with CL.

Additional structure further highlighted source relationships. A secondary cluster (green subclade, BS = 50%, *n* = 11) grouped all ST219 isolates (4 SW, 2 HR) with five HR-associated ST607 isolates, again without clinical representation, reinforcing the close relationship between SW and HR populations. In contrast, ST29 (4 HR, 2 CL) and ST1026-1LV (1 HR, 1 CL) linked HR and CL isolates within shared lineages.

At the finest phylogenetic resolution, direct clustering analysis defined as sister-taxon relationships or terminal groupings connected by minimal branch lengths (red-highlighted clades) showed two cross-source clusters linking SW and HR within the ST25 lineage, though both were supported by short branch lengths rather than strong bootstrap values and no direct SW-CL sister pair was observed. In contrast, HR isolates formed multiple direct cross-source relationships with CL isolates, positioning HR as the most phylogenetically connected source.

Overall, there was generally more self-source clustering (SW = 14/32, HR = 12/32, CL = 16/32) than cross-source clustering. Among the few cross-source clustering, both subclade-level structure and fine-scale clustering indicate that surface water isolates are most closely aligned with those from healthy residents, while also maintaining more limited but detectable connections to clinical lineages. This is reflected in the high proportion of SW isolates sharing sequence types with HR isolates (30/32; 93.8%) compared to CL isolates (3/32; 9.4%), and in the absence of direct SW-CL sister relationships.

#### 3.2.1 Lineage-specific core-genome multilocus sequence typing (cgMLST)

cgMLST was used to quantify allelic relatedness within the four sequence types shared across isolate sources: ST25, ST152, ST219, and ST983 (Fig. 2). Within ST25, comprising 24 surface-water and seven healthy-resident isolates, cross-source distances ranged from 0 to 8 alleles, with a median of 4 alleles. Ten surface-water-healthy-resident pairs had 0 allelic differences across the loci analysed, including AOA9-L55OKP, OEC18KP-L51OKP, and YED1KP-L04JKP.

**Fig. 2:**
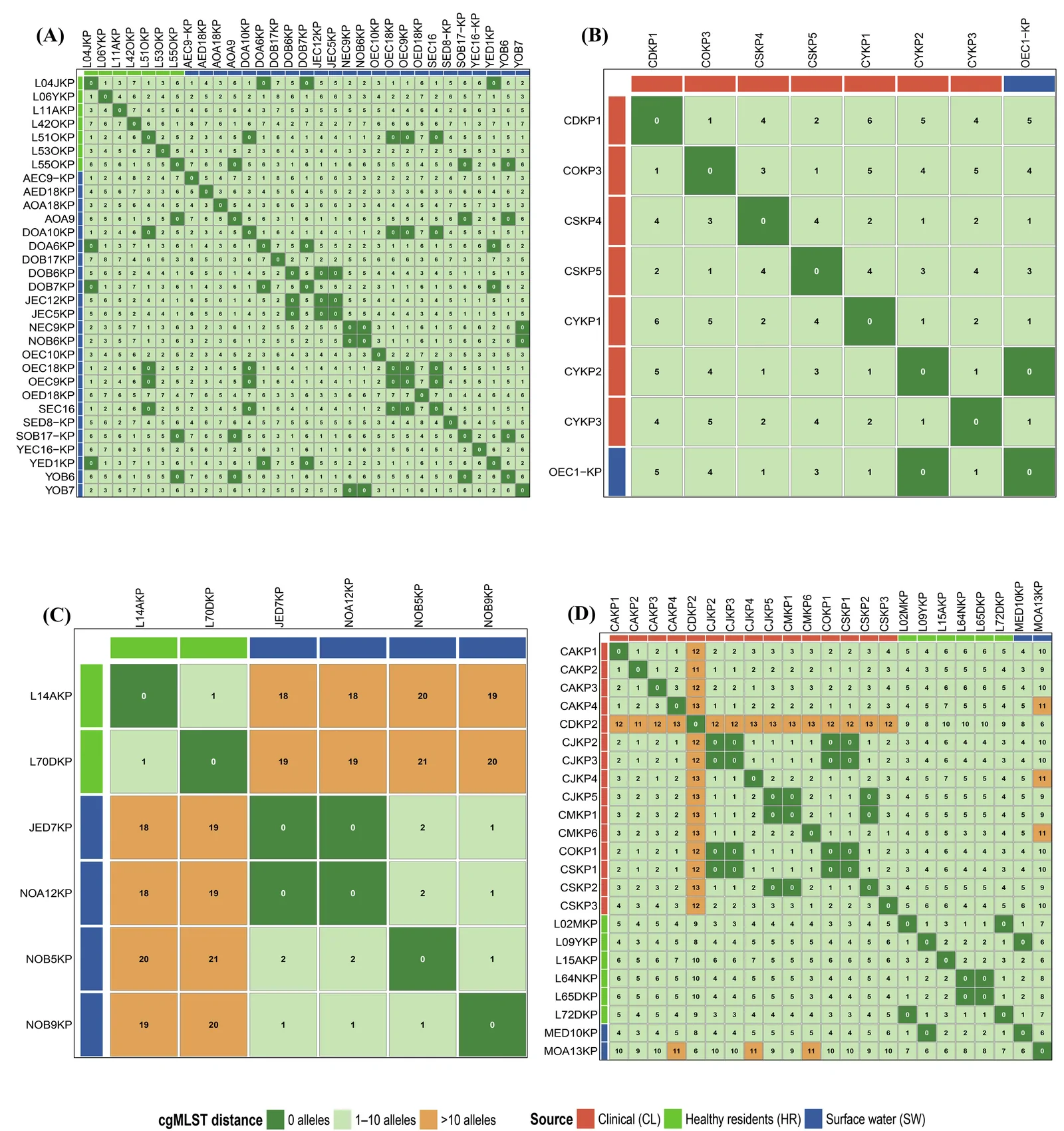
Pairwise cgMLST allelic distances within sequence types shared across isolate sources. Matrices show pairwise allelic differences among (A) ST25 – SW-HR, (B) ST152 – SW-CL, (C) ST219 – SW-HR, and (D) ST983 - HR-SW-CL isolates. Source annotation strips indicate clinical (red), healthy-resident (green), and surface-water (blue) isolates labels. Cell colours represent 0 allelic differences (dark green), 1–10 differences (light green), and >10 differences (orange).

Within ST983, surface-water-healthy-resident isolate distances ranged from 0 to 8 alleles (median, 4), compared with 3 to 11 alleles between surface-water and clinical isolates (median, 7) and 3 to 10 alleles between clinical and healthy-resident isolates (median, 5). The closest ST983 surface-water-healthy-resident isolate pair, MED10KP and L09YKP, had 0 allelic differences, while MED10KP and the closest clinical isolate, CAKP2, differed by 3 alleles.

Within ST152, the single surface-water isolate, OEC1-KP, differed from the seven clinical isolates by 0 to 5 alleles (median, 1); OEC1-KP and CYKP2 had 0 allelic differences. In contrast, ST219 surface-water and healthy-resident isolates differed by 18 to 21 alleles (median, 19), with the smallest distances observed between L14AKP and the surface-water isolates JED7KP and NOA12KP, each differing by 18 alleles.

### 3.3 Detected resistance genes of ESBL *K. pneumoniae* isolates

Across the 96 isolates analysed, a total of 79 antimicrobial resistance genes (ARGs) were identified. β-lactamase genes were the most abundant class, followed by aminoglycoside, then fluoroquinolone, and trimethoprim resistance genes, as seen in Fig. 2a. Macrolide resistance was the least represented.

A core set of 27 resistance genes resistance were shared across all three sources. Among β-lactamases, *bla*_CTX-M-15_ was detected in 27 (84.4%) clinical isolates, 31 (96.9%) healthy resident isolates, and 31 (96.9%) surface water isolates. Similarly, *bla*_TEM-1B_ was highly prevalent across all sources (clinical: 78.1%; healthy residents: 75.0%; surface water: 84.4%). *bla*_OXA-1_ was more frequent in clinical (71.9%) and surface water isolates (84.4%) than in healthy residents (40.6%). Outside the β-lactamase class, among fluoroquinolone-associated determinants, *OqxA* and *OqxB* were highly prevalent in clinical isolates (93.8% and 96.9%, respectively), moderately represented in healthy residents (75% each), and less common in surface water isolates (25% each). In contrast, *bla*_SHV-110_ and *bla*_SHV-81_ were highly prevalent in surface water isolates (75% each), less frequent in healthy residents (21.9% each), and rare in clinical isolates (3.1% each). Similarly, sulphonamide (*sul2*) and trimethoprim (*dfrA14*) resistance genes were widely distributed across all sources, each occurring in >60% of isolates in all groups.

Pairwise analysis revealed the largest number of exclusively shared genes between clinical and healthy resident isolates (*n* = 20), including *aac(3)-IId, aadA2, bla*_CTX-M-3_, *bla*_SHV-172_, *bla*_SHV-187_, bla_SHV-33_, *bla*_TEM-141_, *bla*_TEM-1A_, and multiple additional *bla*_TEM_ variants, as well as *dfrA12, dfrA15, qnrB6*, and *qnrS1*. Surface water and healthy resident isolates shared seven resistance genes not observed in clinical isolates, comprising several *bla*_SHV_ variants (*bla*_SHV-145_, *bla*_SHV-179_, *bla*_SHV-194_, *bla*_SHV-199_, *bla*_SHV-26_, *bla*_SHV-78_, and *bla*_SHV-98_). In contrast, only two resistance determinants were exclusively shared between surface water and clinical isolates: *catA1* and *tet(D).*

Source-restricted resistance genes were also observed. Clinical isolates contained 14 unique genes, including multiple *bla*_SHV_ variants (e.g., *bla*_SHV-103_, *bla*_SHV-11_, *bla*_SHV-13_, *bla*_SHV-40_, *bla*_SHV-56_, *bla*_SHV-70_), as well as *aph(3′)-Ia, catA2*, and *fosA7*. Healthy resident isolates harboured nine unique genes, including *bla*_OKP-B-15_, *bla*_OXA-18_, and several *bla*SHV variants (e.g., *bla*_SHV-65_, *bla*_SHV-75_, *bla*_SHV-94_, *bla*_SHV-96_), in addition to *dfrA17*. No resistance gene was found to be exclusive to surface water isolates. Additional details, including the proportional distribution of resistance genes and chromosomal point mutations across sources, are provided in Supplementary Material S2.

Principal component analysis (PCA) demonstrated substantial overlap in resistome composition across all three sources (Fig. 2C). PERMANOVA analysis identified a statistically significant effect of source on resistome composition (*R*² = 0.075, *p* = 0.003). Chi-square analysis of ARG classes showed significant differences in the distribution of fluoroquinolone, tetracycline, amphenicol, and macrolide resistance genes across sources (*p* < 0.001), whereas β-lactamase, aminoglycoside, sulphonamide, and trimethoprim resistance classes were similarly distributed across sources (*p* > 0.05) (Fig. 3).

**Fig. 3:**
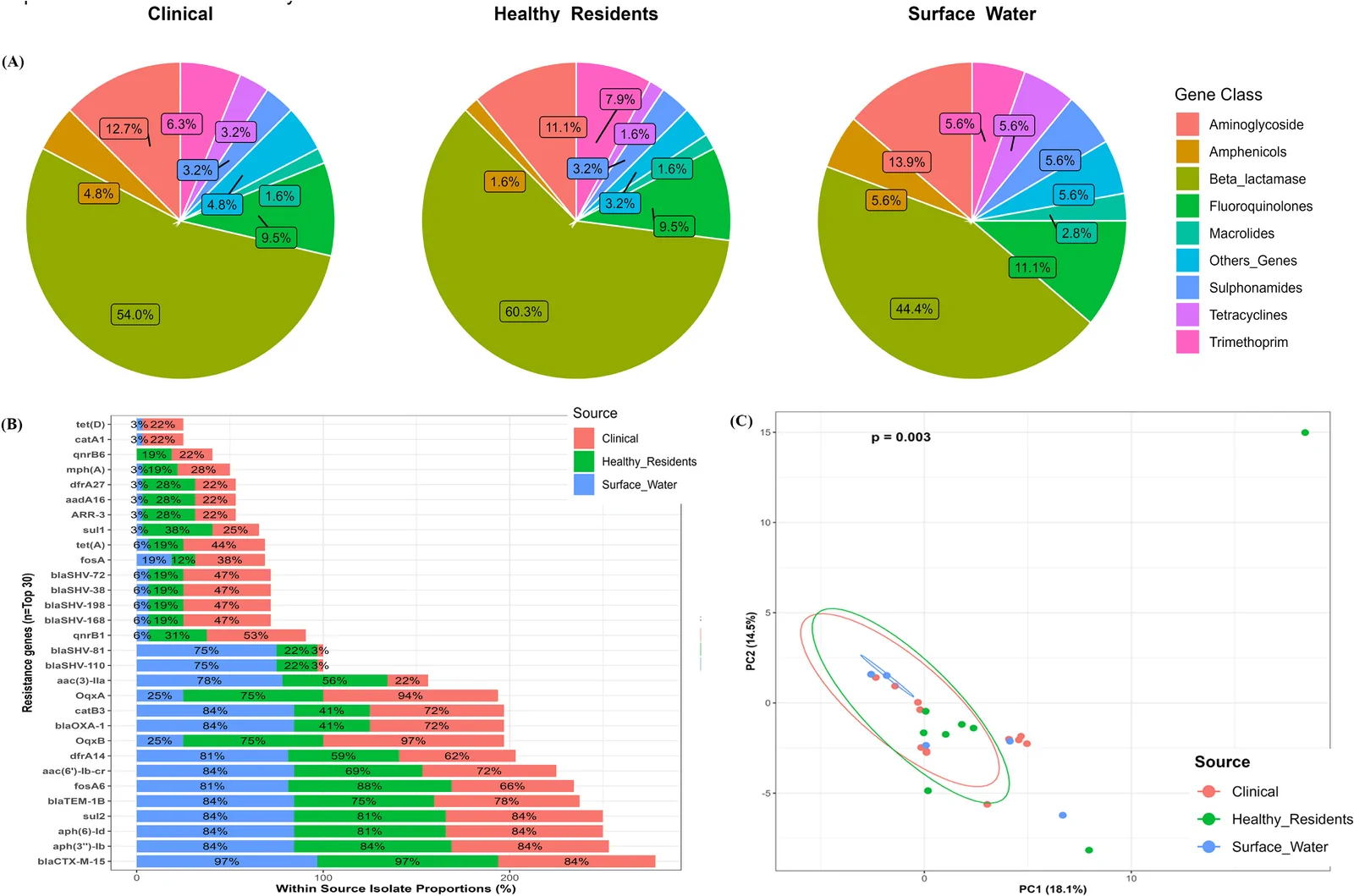
[A] Showing distribution of antibiotic resistance genes across sources by gene classes (B), within sources isolate proportion of top 30 genes, and (C) Principal component analysis (PCA) on the presence–absence matrix of resistance genes detected among the isolates. PCA shows that Clinical (red), healthy residents (green), and surface water (blue) isolates largely occupy the same central region of the ordination space, but resistome composition differs significantly between sources (*p* < 0.05).

#### 3.3.1 Resistance mechanism of ESBL *K. pneumoniae*

CARD analysis revealed 75 distinct resistance genes distributed across seven mechanism categories. Genes including *rsmA, marA, emrA, beaR, adeF, ompA, LptD, K. pneumoniae OmpK37*, and *E. coli AcrAB‑TolC* with *MarR* mutation conferring resistance to CIP and TET were consistently detected in all 32 isolates from each source. The top 60 genes and corresponding isolate counts across sources are shown in Fig. 4A. With the exception of antibiotic target protection, multidrug resistance mechanisms, predominantly antibiotic efflux and antibiotic inactivation, were present in every isolate across all sources (100%). Surface water isolates (*n* = 2, 6.25%) exhibited the lowest representation of antibiotic target protection (Supplementary Material S3/Table 1). Genes belonging to MDR efflux families (ABC, MFS, RND, SMR), reduced permeability, target alteration_PMR, and β‑lactamases were detected in all isolates (100%) from all sources (Supplementary Material/S3/Fig. 4b/c)

**Fig. 4:**
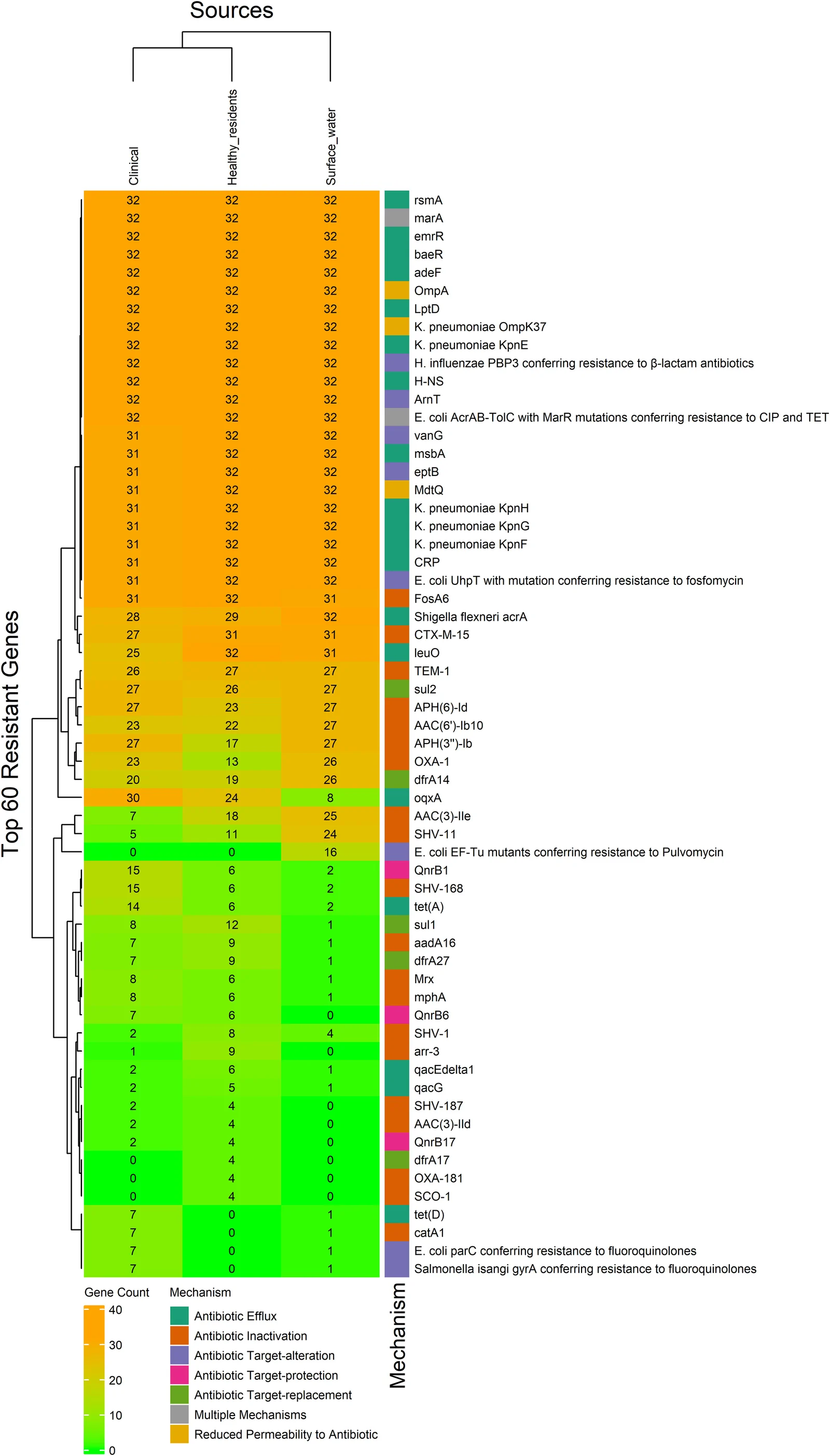
A: Heatmap of resistance genes associated with MDR mechanism across sources.

Resistance mechanism profiles were highly similar across the three sources. All sources showed the same richness value (6), with comparable Shannon diversity values for clinical (H = 1.532), healthy resident (H = 1.540), and surface water isolates (H = 1.472), as well as similar Pielou’s evenness values (0.855, 0.859, and 0.822, respectively). Chi-square analysis showed no significant difference in the distribution of resistance mechanisms across sources (*p* = 0.969).

Heatmap showing the distribution of the top 60 antibiotic resistance genes across Clinical, Healthy Resident, and Surface Water isolates. Genes and corresponding isolate counts are colour-coded from green (low) to red (high), with each row representing a gene. Genes are annotated by resistance mechanism, indicated by colour-coded bars on the right. Efflux, inactivation, and target alteration mechanisms dominate across all sources, while target replacement and reduced permeability are less frequent.

### 3.4 Virulence determinants of ESBL *K. pneumoniae*isolates

Virulence-associated determinants were screened across 96 isolates. The siderophore systems *aerobactin* (*iuc*) and *salmochelin* (*iro*) and the hypermucoidy regulators *rmpA*/*rmpA2* were not detected in any isolates. Fig. 5 (a–f) shows the distribution of respective virulence determinants across sources, while Supplementary Material/S4 details isolate lineages associated with the virulence determinant across sources.

**Fig. 5:**
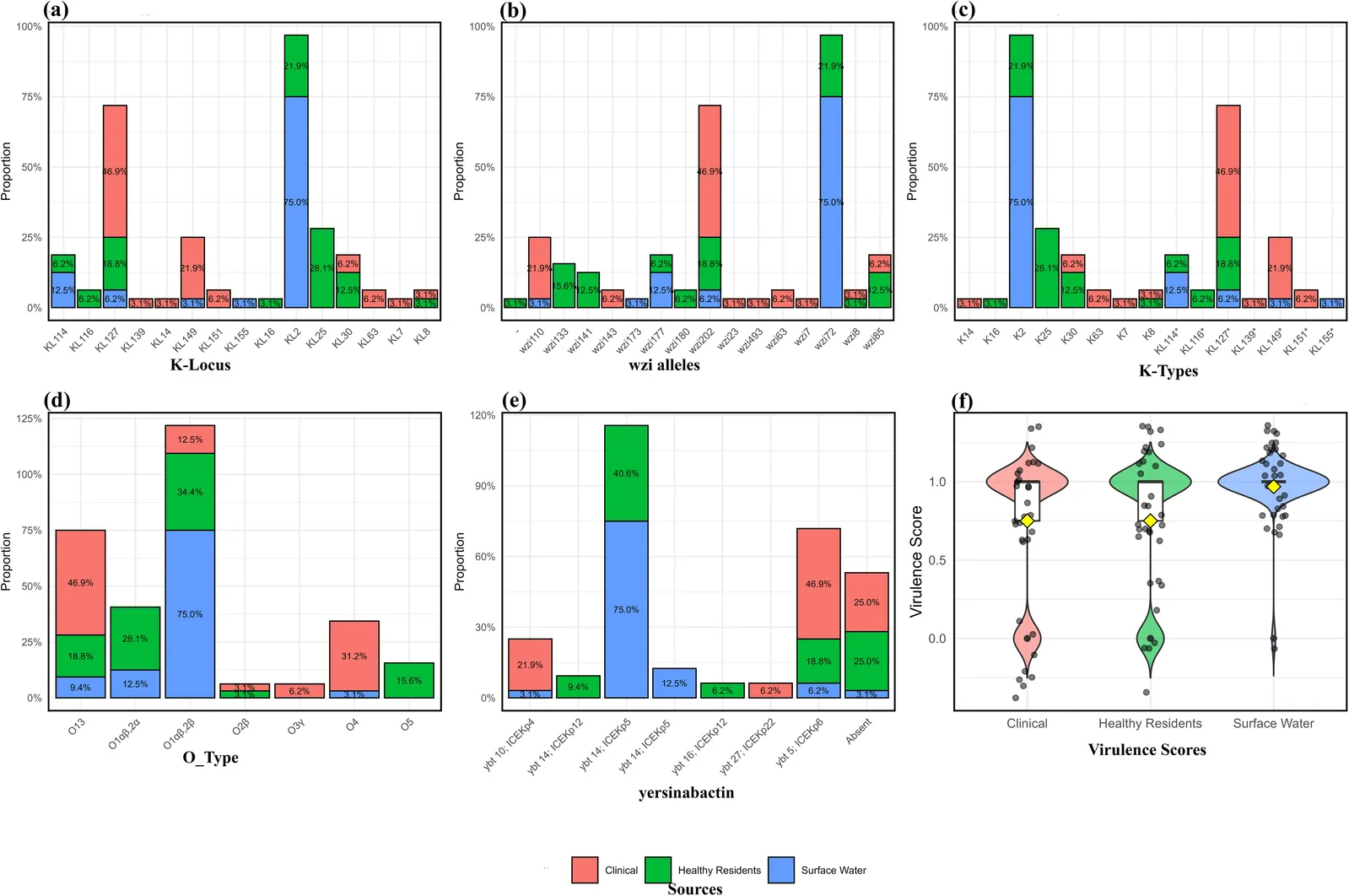
Stacked bar chats of (a) KL-Locus, (b)Wzi alleles, (c) Capsular types, (d) O- Antigens (e)yersiniabactin locus, and (f) virulence score plot of ESBL-Kp Virulence Factors.

Virulence-associated loci, including capsular (K) loci, O-antigen loci, and yersiniabactin-associated *ICEKp* elements, were distributed across a limited number of shared and source-restricted determinants (Fig. 5). Overall, 79/96 isolates (82.3%) carried a virulence score of 1, indicating the presence of yersiniabactin-associated virulence determinants. The only virulence trait shared across all three sources was *KL127/OL13/O13*, corresponding to ST983, detected in 15 CL, 6 HR, and 2 SW isolates, all carrying *ybt5: ICEKp6* (virulence score = 1)

Pairwise sharing was most frequent between surface water and healthy resident isolates. The dominant shared background was ST25/KL2/OL2α.2/O1αβ,2β, present in 24 SW and 7 HR isolates, all carrying ybt14; ICEKp5. A second SW-HR shared background was ST219/KL114/OL2α.1/O1αβ,2α, detected in four SW and two HR isolates, also carrying *ybt14; ICEKp5*. In contrast, the main SW-CL shared background was ST152/KL149/OL4/O4, represented by seven CL and one SW isolate, all carrying *ybt10: ICEKp4*. Healthy resident and clinical isolates also shared ST29/KL30 and ST1026-1LV/KL8, both lacking yersiniabactin (virulence score = 0).

Source-restricted virulence backgrounds were observed mainly in clinical and healthy resident isolates. Clinical-only lineages included ST405/KL151, carrying ybt27; I*CEKp22*, as well as ST37/KL7, ST111/KL63, ST469/KL139, and ST2375/KL14, which lacked yersiniabactin. Healthy resident-exclusive traits included ST133/KL116 with ybt16; *ICEKp12*, and ST17/ST607 with KL25, which variably carried *ybt14* or lacked yersiniabactin. Surface water showed minimal exclusivity, with only ST1380/KL155 lacking yersiniabactin.

Overall, the virulence determinants showed a shared ST983/KL127/OL13 core, broader SW-HR overlap through ST25/KL2 and ST219/KL114, a limited SW-CL link through ST152/KL149, and a small set of source-restricted lineages concentrated mainly in clinical and healthy-resident isolates. At the source level, virulence-positive isolates were detected in 31/32 (96.9%) SW, compared with 24/32 (75.0%) HR and 24/32 (75.0%) CL isolates.

Healthy resident isolates showed the highest virulence-associated diversity and evenness (KL, O loci, O-types, and yersiniabactin; H up to 1.83, J up to 0.83), followed by clinical isolates with intermediate diversity, while surface water isolates had the lowest diversity and evenness across all markers (H ≤ 0.87, J ≤ 0.50). Differences in KL, O loci/types, and yersiniabactin distributions were highly significant across sources (all *p* < 0.001)

## 4 Discussion

Antimicrobial resistance surveillance in sub-Saharan Africa and other low- and middle-income countries remains critically constrained by weak health systems, limited laboratory capacity, and an over-reliance on clinical case detection conditions that render conventional surveillance both incomplete and geographically uneven. Wastewater-based approaches have been proposed as a scalable complement to clinical surveillance, yet their applicability depends on functioning sewage infrastructure that is frequently absent in rural settings (Balcázar, 2025; Punch et al., 2025). In these settings, domestic and community waste drains directly into surface water bodies, making surface water not only a potential reservoir for circulating resistance determinants but also a more contextually appropriate and accessible surveillance matrix (Meradji et al., 2025). To evaluate the potential of surface water as an AMR surveillance proxy, we compared the resistome, virulome, and phylogenetic relationships of ESBL-Kp isolates recovered from surface water, healthy community residents, and nearby healthcare facilities within a spatially connected informal settlement in South Africa to provide local context if AMR profiles in surface water reflect what circulates in the community or clinical setting.

### 4.1 Multilocus sequence typing (MLST) and phylogenomic analysis of isolates

From our findings, ESBL *K. pneumoniae* isolates showed limited overlap in circulating sequence types across three sources, with few pairwise between surface water and either healthy residents or clinical-associated sources lineage-based rather than source-based phylogenetic clustering with isolates from healthy residents and clinical setting. The sequence type, ST983, a lineage associated with hospital infection, was the only notable lineage overlap across all three sources in this study. This is consistent with its previously reported local presence among hospitalised patients and their surrounding hospital environment within the same district (Founou et al., 2019). Beyond local studies, ST983 has been reported among healthy farmers and livestock in a Peruvian One-Health farm study (Benavides et al., 2026), during a neonatal intensive care unit outbreak in Zambia (Nadimpalli et al., 2026), and hospital wastewater in South West England (Gibbon et al., 2021), supporting the broader dissemination across environmental, animal and human niches. Although ST983 isolates in the current study formed a distinct monophyletic clade in the phylogenetic analysis, previous studies have reported a close evolutionary relationship with other clones such as ST17 and ST15 clones associated with bloodstream infections and gastrointestinal colonisation in South Africa (Kopotsa et al., 2020; Salvador-Oke et al., 2024), highlighting the observed overlap as potential of surface water in detecting clinically relevant circulating lineages.

Cross-source pairwise ST analysis showed that surface water isolates were more closely aligned with healthy residents than with clinical isolates. Specifically, 93.8% (30/32) of surface water isolates shared sequence types with healthy residents, primarily through the dominant lineages ST25 and ST219, whereas only 9.4% (3/32) shared sequence types with clinical isolates through ST152. This was further supported phylogenetically, where ST25 and ST219 formed strongly bootstrapped subclades with healthy resident isolates (BS = 100%), compared to the ST152 surface water - clinical association, collectively positioning surface water as more closely aligned with the healthy resident than the clinical isolates. Lineage-specific cgMLST (Fig. 2) provided quantitative support for this pattern: surface-water-healthy-resident pairs differed by 0–8 alleles within ST25 (median, 4; ten pairs with 0 allelic differences) and by 0–8 alleles within ST983 (median, 4). These distances fall within the ≤10-allele range previously used to identify closely related *K. pneumoniae* cgMLST clusters, although such thresholds remain dependent on the cgMLST scheme and epidemiological context (Zhou et al., 2017). Although ST25, ST219, and ST152 have been sporadically reported in South African hospital settings, including paediatric contexts in Durban, Pretoria, Cape Town and Thembisa (Hetsa et al., 2025; Marais et al., 2024; Mbelle et al., 2020; Osei Sekyere et al., 2024), community prevalence remains poorly characterised, likely reflecting surveillance bias toward clinical populations rather than true community absence. While the phylogenetic proximity of surface water to healthy resident isolates is consistent with the informal settlement context of this study where community faecal contamination and waste disposal into surface water are expected, the bootstrapped subclade structure provides genomic-level evidence beyond ST co-occurrence, confirming true clonal relatedness between these compartments. The stronger overlap between surface-water and healthy-resident isolates is consistent with community linkage, but *K. pneumoniae* occurs across human, animal, and environmental reservoirs, and surface water isolates may represent either inputs from human or animal sources or strains established in natural habitats (Rocha et al., 2022). Notably, ST152 has previously been documented in both carriage and clinical isolates locally (Founou et al., 2019; Masalane et al., 2025), and recovered from a wastewater treatment plant and its receiving water body in the same geographical area (Ramsamy et al., 2022), corroborating its role as an ecologically versatile lineage bridging carriage, environmental, and clinical compartments and lending direct precedent to our findings.

Notwithstanding the cross-source overlaps, self-source clustering was the predominant pattern overall, with 14/32 (43.8%) of surface water, 12/32 (37.5%) of healthy residents, and 16/32 (50.0%) of clinical isolates forming within-source groupings, suggesting that each source retains a degree of ecological distinctiveness despite shared lineages. This was most evident among healthy resident and clinical isolates, where several maximally supported (BS = 100%) exclusively within-source subclades were identified. Healthy residents formed self-exclusive clades encompassing ST17, ST607, and ST133 lineages, none of which were recovered from surface water or clinical samples, while clinical isolates similarly formed exclusive, well-supported groupings within ST405 and ST111. Surface water likewise harboured its own exclusive sequence type, with ST1380 absent from both healthy resident and clinical ST repertoires. Notably however, despite its ST-level exclusivity, ST1380 clustered phylogenetically within the healthy resident neighbourhood, grouping with ST17 isolates at a bootstrap of 84%, indicating that even the surface water-restricted lineage maintains greater genomic affinity to the community compartment than to clinical isolates. The absence of surface water representatives from the healthy residents and clinical exclusive clades, combined with the community-directed phylogenetic positioning of its own exclusive lineage, argues against surface water being a passive mirror of either source, and instead supports its role as a selective environmental reservoir that captures specific shared lineages, particularly ST25, ST219, and ST983, while remaining ecologically distinct in its overall population composition. Overall, the combined MLST and phylogenomic evidence suggests that surface water in this setting does not fully recapitulate the community or clinical *K. pneumoniae* population, but instead captures a meaningful subset of circulating lineages, with its closest phylogenetic relationship being to healthy residents rather than clinical isolates.

### 4.2 Antibiotic resistance genes (ARGs) resistance mechanism of ESBL-Kp isolates

Comparative resistome analysis of the study isolates (Fig. 3) revealed a substantial shared core set of determinants across clinical, healthy resident, and surface water sources, indicating the interconnected circulation of resistance determinants across sources. This was reflected by the detection of 27 ARGs common to all sources, including the globally disseminated ESBL gene *bla*_CTX-M-15_, *bla*_TEM-1B_, and non-β-lactam determinants such as *sul2* and *dfrA14*, with no significant difference in the distribution of resistance gene categories such as β-lactamase, aminoglycoside, sulphonamide, and trimethoprim resistance across sources. The high prevalence of *bla*_CTX-M-15_ and *bla_TEM-1B_* across all three sources complements earlier reports on increased prescription and usage of β-lactam antibiotics such as penicillins and cephalosporins in South Africa (Alabi and Essack, 2022) while the ubiquity of *sul2* and *dfrA14* similarly mirrors reported widespread co-trimoxazole (trimethoprim-sulfamethoxazole) use for urinary tract infection management and HIV prophylaxis in the South Africa (Fourie et al., 2021; Nel et al., 2023). Consequently, recent local studies on Klebsiella species from clinical (Hetsa et al., 2025; Masalane et al., 2025; NDoH, 2024), community (Ogunbosi et al., 2020), and environmental (Motlhaping et al., 2026; Richter et al., 2021) sources have noted high prevalence of these genes. This shared genetic determinant was matched by a highly conserved resistance mechanism profile, with multidrug efflux systems detected universally across all sources (Fig. 4A). The substantial overlap observed in the PCA (Fig. 3C) and the small but statistically significant PERMANOVA effect size ($R^{2}=0.075$) indicate a modest source-associated shift rather than strong biological separation, with most resistome variation not explained by source. However, this overall similarity does not preclude source-specific variation in individual resistance traits.

Despite this common backbone, several source-dependent patterns indicate ecological differentiation of the resistome. Clinical isolates were enriched for fluoroquinolone-associated determinants (*oqxA, oqxB*), likely reflecting stronger antibiotic selection pressure in the clinical setting from high-watch antibiotic category prescribing (Mthombeni et al., 2023; Spinickum et al., 2024), and the markedly lower prevalence of these determinants in surface water isolates suggests weaker maintenance in the aquatic environment, possibly due to reduced antimicrobial selection and differing ecological trade-offs rather than a fundamental ecological discontinuity between sources (Larsson and Flach, 2022). In contrast, surface water isolates showed marked enrichment of multiple *bla*SHV variants, consistent with the accumulation of diverse resistance lineages introduced by mixed anthropogenic inputs, including domestic wastewater, community faecal discharge, and hospital effluent, each potentially contributing distinct *bla*SHV alleles. Healthy resident isolates displayed an intermediate profile, sharing the greatest number of exclusive ARGs with clinical isolates, suggesting that asymptomatic carriage populations may function as an epidemiological bridge between hospitals and the wider environment. The significant between-source differences in fluoroquinolone, tetracycline, amphenicol, and macrolide resistance classes, contrasted with the similar distribution of β-lactamase, aminoglycoside, sulphonamide, and trimethoprim genes, further indicate that while the core resistome is conserved, accessory resistance traits remain responsive to local antimicrobial selection pressures.

From a surveillance perspective, surface water partially mirrored the human-associated resistome but did not fully reproduce the community and clinical relationship. Notably, no ARG was unique to surface water isolates, whereas both clinical and healthy resident isolates harboured source-restricted genes, suggesting that ESBL *K. pneumoniae* from surface water profiles largely reflect redistributed community and clinical resistance rather than an independent reservoir of novel determinants. However, the limited overlap of exclusive genes between surface water and clinical isolates indicates that aquatic surveillance alone would underestimate certain clinically relevant resistance signatures. Notably, surface-water-only surveillance would not have captured 14 resistance genes detected only in clinical isolates and nine detected only in healthy-resident isolates or several sequence types absent from surface water, including ST17, ST29, ST111, ST133, ST405, ST469, ST607, ST2375, and ST3216. Nevertheless, the consistent detection of widely circulating ARGs in surface water supports its role as a complementary surveillance matrix, particularly for population-level monitoring of dominant resistance trends. Surface water should therefore be considered an adjunct, rather than a substitute, within integrated One Health AMR surveillance frameworks.

### 4.3 Virulome of ESBL – *K. pneumoniae* across sources

None of the isolates carried *aerobactin* (*iuc*), *salmochelin* (*iro*), or the hypermucoidy regulators *rmpA*/*rmpA2*, confirming the absence of the hypervirulent *Klebsiella pneumoniae* (hvKp) pathotype across all sources. Virulence was instead dominated by yersiniabactin, with 82.3% of isolates carrying ICEKp-encoded ybt loci (virulence score = 1). This is consistent with a South African finding showing that multidrug-resistant clinical *K. pneumoniae* exhibit substantial capsular diversity but lack hypervirulence genes, reflecting predominantly classical lineage (Mbelle et al., 2020). These findings indicate a population in which virulence is primarily mediated by iron acquisition rather than hypervirulence-associated plasmids. However, variation in yersiniabactin lineages and capsular backgrounds across sources indicates that the virulome is not uniform and remains source-structured. Similar patterns have been reported elsewhere, where overlap between clinical and environmental *K. pneumoniae* populations coexists with uneven distribution of virulence determinants, with yersiniabactin widespread while *iuc, iro*, and *clb* are largely restricted to clinical lineages (Rocha et al., 2022).

The strongest inter-source relationship was observed between surface water and healthy resident isolates, driven primarily by ST25/KL2/*ybt14; ICEKp5* and, to a lesser extent, ST219/KL114/*ybt14; ICEKp5*, whereas overlap between surface water and clinical isolates was limited. Given that ST25 is a clinically documented (Hetsa et al., 2025; Olwagen et al., 2025; Zhao et al., 2022) but epidemiologically heterogeneous lineage, its dominance in surface water supports the interpretation that aquatic isolates capture broader community-associated backgrounds rather than hospital-enriched populations. This interpretation is reinforced by the near-universal detection of virulence-positive isolates in surface water (96.9%), coupled with markedly reduced diversity and evenness relative to healthy residents. Together, these patterns are consistent with clonal enrichment of a limited number of successful lineages in the aquatic environment, rather than broad representation of the underlying human virulome.

Source-restricted lineages were concentrated in clinical and healthy resident isolates, while surface water showed minimal exclusivity. The significantly lower diversity observed in surface water compared to healthy residents indicates that, although dominant community-associated lineages are captured, the broader virulome diversity present in the human population is not. Consequently, surface water does not provide a comprehensive proxy for either the clinical or the community virulome composition. Rather, it reflects a restricted subset of circulating lineages, with stronger alignment to community-associated backgrounds. Surface water surveillance may therefore be informative for detecting dominant or emerging lineages but should be interpreted as complementary to, rather than a substitute for, direct human sampling within integrated One Health surveillance frameworks.

This study has some limitations to consider when interpreting findings. First, sampling one informal settlement and one river catchment limits generalisability. Findings are thus most applicable to settings with comparable hydrology, sanitation, land use, climate, and healthcare connectivity. Future studies should include multiple communities and river catchments spanning contrasting conditions to test whether these patterns hold more broadly. Second, all 32 available clinical genomes were analysed, whereas only balanced, month-stratified subsets of 32 isolates were selected from the larger surface-water and healthy-resident isolate pools. Although this improved comparability, the subsets may not have captured the full genomic diversity present in the larger pools, and alternative random subsets could have produced different estimates of source overlap and diversity, particularly for rare sequence types, low-frequency resistance or virulence genes, and source-restricted features. Future studies should include larger isolate numbers, repeated resampling, and extended seasonal coverage to assess the stability of the findings, formally evaluate temporal changes in resistance genes and sequence types, and determine the plasmid or chromosomal location and genetic context of key resistance determinants. Thirdly, isolate-level genotype-phenotype concordance was not assessed, limiting evaluation of how reliably detected resistance genes predicted phenotypic resistance. Future studies should integrate paired antimicrobial susceptibility and genomic analyses to quantify this concordance. Finally, livestock, wildlife, agricultural, stormwater, and other environmental inputs were not sampled, precluding formal source attribution. Parallel sampling of these inputs, combined with hydrological and microbial source tracking, would strengthen attribution, and operational research should compare cost, turnaround time, sensitivity, and surveillance value.

## 5 Conclusion

This study evaluated whether surface water can serve as an alternative matrix for human AMR surveillance by comparing the resistome, virulome, and phylogenetic relatedness of ESBL-producing *K. pneumoniae* from surface water, healthy residents, and clinical sources. The findings show that surface water can provide useful surveillance information on ESBL-producing *K. pneumoniae* circulating within the study setting, particularly by detecting dominant community-associated lineages and commonly shared resistance determinants. However, surface water did not fully represent the genomic, resistance, or virulence diversity observed among healthy-resident and clinical isolates.

Surface-water isolates were more closely aligned with healthy-resident isolates than with clinical isolates, indicating that surface water in this setting more closely reflected community-associated carriage than clinical infection. Although shared resistance and virulence features were detected across sources, source-specific lineages, resistance determinants, and virulence profiles were underrepresented or absent in surface water.

Surface water may therefore provide a useful complementary surveillance matrix in communities where sewer-based wastewater surveillance is unavailable and community-level AMR data are limited. Its findings should be interpreted alongside clinical and community surveillance data within an integrated One Health AMR surveillance framework.

## 6 Declaration of generative AI and AI-assisted technologies in the manuscript preparation process

Generative AI and AI-assisted writing tools, including ChatGPT and Grammarly Premium, were used to support language editing, improve clarity and coherence of writing, assist with refining, summarise and clarify complex scientific concepts. Generative AI was not used for data generation, statistical analysis or reference management. All AI-assisted content was critically reviewed, edited, and verified for scientific accuracy, relevance, and consistency with the study findings.

## CRediT authorship contribution statement

**Etando Ayukafangha:** Writing – review & editing, Writing – original draft, Methodology, Investigation, Formal analysis, Data curation, Conceptualization. **Joshua Mbanga:** Writing – review & editing, Writing – original draft, Visualization, Validation, Supervision, Methodology, Data curation. **Arshad Ismail:** Writing – review & editing, Writing – original draft, Resources, Investigation, Formal analysis, Data curation. **Sabiha Y. Essack:** Writing – review & editing, Writing – original draft, Visualization, Validation, Supervision, Resources, Project administration, Methodology, Investigation, Funding acquisition, Data curation, Conceptualization. **Akebe Luther King Abia:** Writing – review & editing, Writing – original draft, Visualization, Validation, Supervision, Methodology, Investigation, Formal analysis, Data curation, Conceptualization.

## Declaration of competing interest

S.Y.E. is a chairperson of the Global Respiratory Partnership and a member of the Global Hygiene Council, both supported by unrestricted educational grants from Reckitt (Pty.) Ltd., UK. All other authors have no competing interests.

All authors have read, understood, and complied as applicable with the statement on “Ethical responsibilities of Authors” as found in the Instructions for Authors.

## Data Availability

Data will be made available on request.

## Appendix Supplementary materials

Image, application 1
